# Health and Welfare Benefits of Computerized Cognitive Enrichment in California Sea Lions (*Zalophus californianus*) at the US Navy Marine Mammal Program

**DOI:** 10.3390/ani14071120

**Published:** 2024-04-06

**Authors:** Kelley Winship, Abby McClain, Amber Ramos, Jennifer Dunham, Mark Xitco

**Affiliations:** 1 National Marine Mammal Foundation, 2240 Shelter Island Dr., Suite 200, San Diego, CA 92106, USA; 2 U.S. Navy Marine Mammal Program, Naval Information Warfare Center Pacific, Code 56700, 53560 Hull Street, San Diego, CA 92152, USA

**Keywords:** cognitive enrichment, California sea lion, computerized task, pinniped welfare

## Abstract

**Simple Summary:**

This study presents evidence that the introduction of a cognitively challenging computerized system for US Navy California sea lions during positive-reinforcement training sessions was related to an increase in session participation, measured through the consumption of the offered diet each day. Additionally, a reduction in the number of days animals were clinically ill was observed, suggesting an increase in sea lion welfare following the implementation of computerized enrichment.

**Abstract:**

Cognitive enrichment is becoming more prevalent in professional marine mammal facilities. Research with dolphins has suggested that such enrichment provides more welfare benefits than enrichment that does not incorporate cognitive challenge. However, there is little research supporting the use of cognitive enrichment as a means to improve the welfare of sea lions. Recently, a novel form of technological cognitive enrichment, the Enclosure Video Enrichment (EVE) system, was introduced to a population of California sea lions at the US Navy’s Marine Mammal Program as a means to enhance welfare. Two of the initial focal animals introduced to EVE were selected based on their health history and the possible benefits of cognitive enrichment in improving health measures. To evaluate this, information regarding the animals’ consumption of their offered diet was compared to other animals in the population of similar age and the absence of a similar health history. Subsequently, the total diet consumption of the targeted animals was evaluated in the two years prior to the introduction to EVE and compared to the total diet consumption during the initial 2 years of regular EVE sessions. There was a significant decrease in the number of days in which the sea lions did not consume their entire offered diet in the two years after implementing regular EVE sessions, an increase in participation and performance of voluntary husbandry behaviors, and a reduction in the number of days animals were clinically ill. This study provides evidence of cognitive enrichment as a management tool to improve animal health and welfare as well as performance in training sessions.

## 1. Introduction

The welfare of professionally managed animals has experienced a surge in research interest in recent years. The original welfare model of the Five Freedoms has been updated to include the Five Domains, which includes the impact that human behavior and modifications have on welfare [1]. Animal welfare initially focused on the physical health of the animal through monitoring parameters such as disease, lesions, and body condition [2]. The psychological welfare of animals, evaluated using the presence of stereotypic and maladaptive behaviors, as well as general behavioral profiles of animals (e.g., sociality, lethargy), has become an important addition, motivated in part by public concern for the wellbeing of zoo animals (e.g., [3,4,5,6,7,8,9]) but also by the demonstrated relationship between psychological and physiological health in animals. For example, stress and its related hormones are known to negatively impact health and welfare in many species (e.g., [10,11,12,13]).

One method for enhancing the psychological and physical health of professionally managed animals has been the incorporation of positive reinforcement training for enrichment and healthcare [14,15,16,17,18,19,20,21]. For marine mammals in particular, positive reinforcement training has been a consistent means of managing the health of animals in educational, research, and military settings [22,23,24]. Such training allows for safer outcomes in various species (e.g., [25,26,27,28]), and the large size of many marine mammal species has encouraged the incorporation of such training to minimize animal stress and injury from restraint (e.g., [20,22,29]). Through positive reinforcement training, such behaviors can become a stress-free and reliable means of collecting important information [22] that forms the cornerstone of successful preventative medical programs for animals [30]. Most marine mammal welfare research has centered on dolphins, exemplified by the development of the C-Well welfare assessment [31] and the multi-institutional Cetacean Welfare Study [24,32,33,34,35,36,37,38,39]. Progress with pinnipeds has lagged [40].

Recognizing animals’ intrinsic motivation for exploration and interaction with novel stimuli may be key for developing new avenues for enrichment. According to Mench [41], animals exhibit a preference for engaging with unfamiliar environments and objects, a behavior indicative of information seeking that is likely adaptive in natural habitats. These actions, which include contrafreeloading and exploratory behavior, imply a reward mechanism intrinsic to the animals. The lack of such engagement opportunities is associated with negative psychological states, including boredom, depression, and anxiety, underscoring the critical role of environmental enrichment in enhancing animal welfare [42]. This necessitates the integration of diverse and stimulating environments in managed care to fulfill animals’ exploratory needs, thereby improving their overall well-being and reducing negative mental health outcomes [43,44]. This aligns with the broader objective of promoting enriched habitats that support natural behaviors and psychological health of animals. 

Additionally, introducing more cognitively complex enrichment (see [45]) for marine mammals has been proposed, with an emphasis on maintaining effectiveness through enrichment variability [46] to avoid habituation (e.g., [47]), despite limitations on staff availability [48]. While enrichment programs are largely successful in reducing stereotypic or targeted maladaptive behaviors (see [48,49]), there has been less reporting on how an animal’s general physical health can be positively impacted longer term, or how individual differences (e.g., [50,51,52,53,54]) might play a role in enrichment effectiveness.

Technology may provide solutions to these issues for sustainable and effective marine mammal enrichment as it has for a variety of zoologically housed terrestrial species (see [55]), from playback of bird sounds to motivate hunting behaviors in African leopards (*Panthera pardus*; [56]) to encouraging interaction with the environment through randomized access to food in magnetized boxes in tigers (*Panthera tigris altaica*; [57]). In both instances, the technology was successful in reducing stereotypic behaviors in the focal animals [56,57]. Further development of technological interfaces has also allowed animals to control cursors, resulting in the ability to test cognition and cognitively enrich animals that are trained to interact with the systems, especially non-human primates (e.g., [58,59,60,61,62]). The addition of choice and control, from directly controlling a game object to allowing animals to select the computerized task to engage in, may also provide additional welfare benefits [60,62,63]. For example, Capuchins (*Cebus apella*) prefer to complete tasks that they select, even if the computer chooses tasks based on the animals’ preference [59]. Other forms of technological enrichment (e.g., video clips) have also been successfully implemented to reduce maladaptive and stereotypic behaviors in primates [64], in cetaceans such as bottlenose (*Tursiops truncatus*) and rough-toothed dolphins (*Steno bredanensis*; [54]), and in a singly-housed killer whale (*Orcinus orca*; [65]).

Anecdotes of marine mammal species reducing participation in training sessions and reducing consumption of their diet prior to diagnosis of illness have been present for decades [66] and are common markers for illness in other species [67,68]. The willingness to participate (WtP) in positive reinforcement training sessions has been used as a measure of animal welfare and is predictive of a decline in health status in several species, although only severe disease has been accurately predicted. Clegg and colleagues [69] found a significant relationship between WtP in bottlenose dolphins and the percentage of offered diet consumed, as well as a significant relationship between a veterinarian determined health score and the percentage of diet consumed by the animals. Diet consumption in California sea lions (*Zalophus californianus*) fluctuates seasonally related to environmental factors such as rising or falling temperatures [70], so changes in interest in food may not always indicate illness.

Historically, pinniped enrichment programs have involved objects [46,71,72], puzzle feeders [73], training sessions [74], and novel scents [75]. Though a large amount of research has been conducted evaluating sea lion cognition (see [76]), cognitively challenging enrichment has not been as extensively reported as for dolphins (e.g., [77,78]). Additionally, there is little information correlating potential health improvements to the introduction of a computerized cognitive enrichment system in marine mammals and how this might impact animals with a history of inconsistent participation in positive reinforcement training sessions and completion of their offered diet. The Enclosure Video Enrichment (EVE) system was developed at the US Navy Marine Mammal Program (MMP) to provide technological enrichment and cognitive testing for Navy sea lions [79] and bottlenose dolphins. EVE was provided to enhance welfare by providing additional enrichment and cognitive stimulation following free-release sessions, while also providing a means to more effectively evaluate comparative cognition.

The introduction of more challenging games on EVE has been shown to have a positive impact on gameplay efficiency as well as interest in the system in this population of California sea lions [80]. The goal of this study was to examine the impacts of this cognitively challenging system on animal health and interest in participation in training sessions when it was incorporated as a regular session type.

## 2. Materials and Methods

This study was conducted at the Naval Information Warfare Center (NIWC) Pacific in San Diego, California. Sea lions at the MMP were housed in 9 × 9 m^2^ floating sea pens with attached haul-out areas. Animals are housed in groups that vary in composition daily or biweekly. While combinations can vary between the individuals in San Diego, at least two and no more than five individuals are grouped together. The study subjects were housed with conspecifics not included in the study and with each other at varying times throughout the study period. The MMP is accredited by The Association for Assessment and Accreditation of Laboratory Animal Care (AAALAC) and follows the standards of the U.S. Public Health Service Policy on the Human Care and Use of Laboratory Animals and the Animal Welfare Act. The data collection for this study was also approved under the MMP Institutional Animal Care & Use Committee (IACUC) Protocol #139-2020 and was reviewed by the Navy Bureau of Medicine and Surgery as Navy Research Database #1245.

Training sessions for sea lions at the MMP cover a broad spectrum of activities, including shaping of new husbandry behaviors, open-water tasks, or maintenance of previously acquired behaviors. The session length varies from five-minute interactions within the sea lion’s home enclosure to complex sessions that can last several hours outside their primary enclosure. EVE sessions are generally conducted within the sea lions’ home enclosure, where the system is set up and visible to the animal prior to them being asked to separate from conspecifics and participate in a session with a trainer. Each successful completion of a trial during an EVE session is rewarded with fish and verbal praise. The quantity of fish fed varied across trials; however, sea lions generally consumed between 25 and 50% of their daily diet in these sessions. The percentage was comparable with the amount fed during most other training sessions, while open-ocean sessions often consist of the consumption of a larger portion of the animals’ diet.

Initially, EVE was introduced as the focal task of the sessions in which it was used. However, as the sea lions became more proficient with EVE, it was implemented as an immediate reinforcer for completing more challenging tasks, such as voluntary participation in medical procedures like blood draws or walking out-of-pen to an unfamiliar location.

At the end of each workday, animal care staff electronically documented details regarding the diet offered versus what was consumed by the animals, alongside notes on the animals’ health, appearance, physical activity, and overall performance. The archived diet and session availability data from four adult male sea lions were obtained from the MMP records office for the relevant dates for each animal. Two focal sea lions had a history of stereotypy resulting in them not completing their offered diet, while a third animal with no similar history was taught to interact with the system. One additional male with no exposure to EVE during this time was also used as a control subject of a similar age to the focal animals. Data from 2018 to 2022 were used and divided into two-year time periods. The time periods varied by date for each animal based on the start date of EVE exposure but occurred between 22 July 2018 and 9 September 2022. Two years (730 days) pre-EVE exposure (Time Period 1) and the first two years (731 days due to leap year) of regular EVE sessions (Time Period 2) were used for three animals, while corresponding numbers of days were obtained for the animal that was not participating in EVE during that time period (Table 1). The average EVE session lengths for ANK and REX during the data collection time period were under 15 min, with SLD’s sessions averaging closer to 20 min [79].

Interest in participation was determined through the completion of the animals’ offered diet, which was used as a proxy for general motivation in training sessions. Sea lions are offered their diet daily throughout several sessions and may be able to supplement their diet through foraging in their netted enclosures in San Diego Bay. Offered diets are determined by the animal’s veterinarian and lead government trainer based on season, age, and size, with changes occurring throughout the year to compensate for differences in caloric requirements resulting from seasonal environmental temperature changes to maintain a healthy body weight. Days in which the incomplete consumption of the offered diet (ICOD) occurred were compared between EVE and non-EVE individuals during the different time periods, as well as individuals that have a history of incomplete diet consumption.

Days in which the animals were provided more than their initial planned diet were not included in the data set. Dates during which animals were considered to have an acute illness, thus impacting their interest in sessions and food due to related symptoms (e.g., gastrointestinal distress), were also not used in the analysis of ICOD days. Animals were considered to be acutely ill by an attending veterinarian if their hematological and/or biochemical blood results showed values outside of the normal population reference ranges (MMP in-house reference ranges) and/or they were prescribed antimicrobials, anti-nausea medications or were provided other forms of supportive care (e.g., subcutaneous fluids, oral fluids, etc.) that may affect their appetite. Additionally, any dates in which human error may have resulted in inaccurate (e.g., a decimal value not normally used in diet measurements was entered) diet recordings were also not used. The total number of days analyzed for each animal is shown in Table 2. 

**Figure 1:**
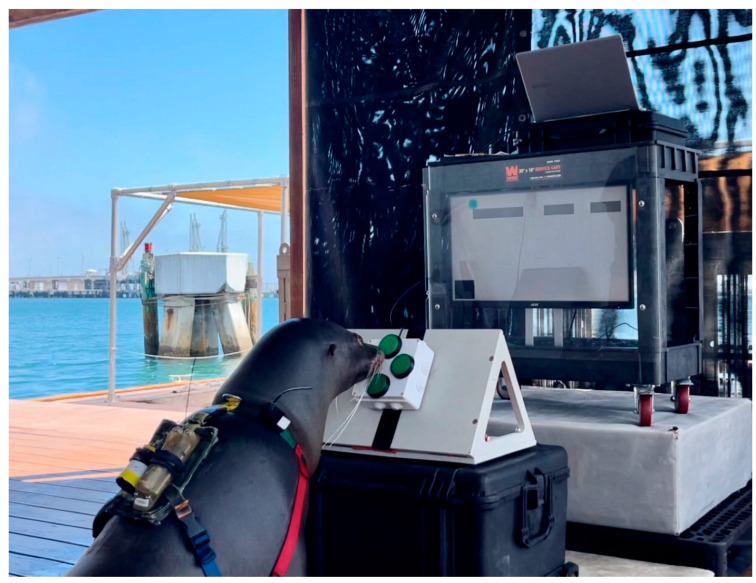
A sea lion playing the MAZE Training Game during an out-of-pen session. The controller and cart are raised for better visibility by the sea lion, and the monitor presents the games played through the laptop on the top of the cart. The sea lion moves the blue circle cursor across a grey platform to contact the black square at the bottom left side of the screen.

EVE consists of a monitor mounted to a utility cart, and a four-button directional controller (Figure 1). EVE was presented to the animals (ANK, REX and SLD) during trainer supervised sessions, and during the two-year time period the animals successfully learned to control a cursor (see [79] for a detailed description of the setup and training) and began learning more challenging tasks that incorporated failure [80]. The sea lions were fed a portion of their daily diet during these sessions for successful responses.

A Chi-square test was used to compare the number of ICOD days by health status and by time period for individuals. A nested ANOVA was used to assess the change in average difference (Δ_D_) between the offered and consumed diets, in lbs., between time periods based on animal health status (i.e., focal or control). Statistical analysis was completed in SPSS Version 21.

## 3. Results

### 3.1. Incomplete Diet Consumption Day Analyses

**Figure 2:**
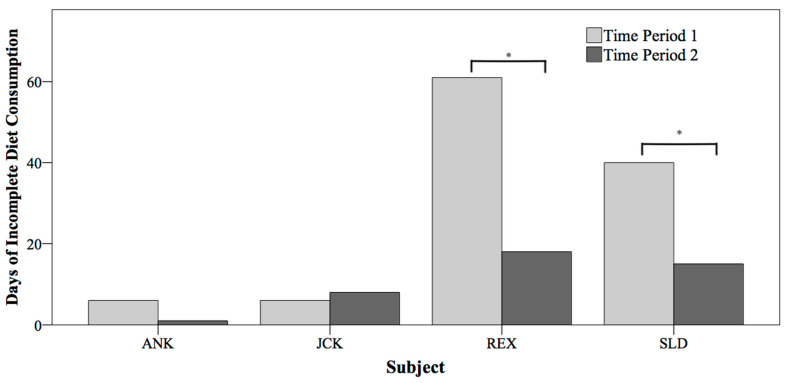
The number of ICOD days between individual and two-year time period. There was a statistically significant decrease in ICOD days between time periods for the two focal animals, REX and SLD, marked by asterisks.

Focal animals had significantly less ICOD days in Time Period 2 (n = 33) than in Time Period *1* (n = 101, X^2^ = 37.21, *p* < 0.001; Figure 2). The significant reduction in ICOD days was seen in both REX (X^2^ = 24.77, *p* < 0.001) and in SLD (X^2^ = 12.57, *p* < 0.001) between the Time Periods (Figure 2). There was no significant difference in ICOD days for the control animals (X^2^ = 0.37, *p* = 0.543).

### 3.2. Diet Consumed Analyses

**Figure 3:**
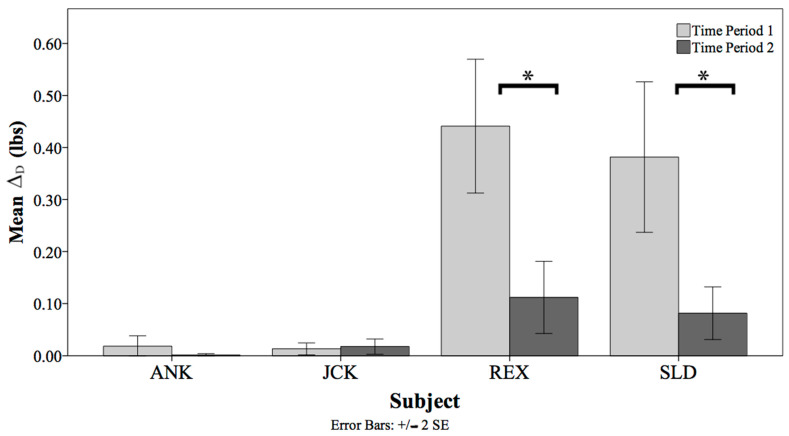
The mean Δ_D_ between the two time periods for each animal. Δ_D_ is calculated by subtracting the consumed diet from the offered diet. Significant differences are noted by asterisks.

A nested ANOVA was used to compare Δ_D_ between the fixed factor of Time Period and the random factor of Health Status. There was a significant difference in Δ_D_ across the Time Periods between the Health Statuses (F(1) = 26.78, *p* < 0.001, η^2^ = 0.55; Figure 3). 

REX had a significant decrease in Δ_D_ between Time Period 1 (M = 0.44) and Time Period 2 (M = 0.11), t(1432) = 4.505, *p* < 0.001. SLD also demonstrated a significant decrease in Δ_D_ between Time Period 1 (M = 0.38) and Time Period 2 (M = 0.08), t(1420) = −3.92, *p* < 0.001. There was no significant difference in Δ_D_ for ANK or JCK across the two time periods. 

### 3.3. Number of Clinically Ill Days

There was a decrease in the number of clinically ill days for both REX (5 days in Time Period 1 and none in Time Period 2) and SLD (25 days in Time Period 1 and 1 day in Time Period 2). JCK had no clinically ill days in Time Period 1 but had two bouts of illness totaling 19 days in Time Period 2. ANK did not have a single clinically ill day during the four years examined in this study.

## 4. Discussion

This study provides evidence that the introduction of a technological enrichment system in the form of cognitively challenging computerized tasks may serve as a means to improve animal participation in sessions, and subsequently animal health. There was a significant reduction in ICOD days of the focal individuals between the two-year time period prior to EVE and the time period of offered EVE sessions. The lack of significant difference of ICOD days between the time periods for the control animals suggests that the implementation of EVE in the training and management plan of the focal animals had a strong impact on the improvement of the animal’s health and welfare, and it was less likely that this improvement was due to some other environmental or management change that impacted the wider population of Navy sea lions. Additionally, there was a small but statistically significant decrease in Δ_D_ between the two time periods for the focal animals, even with an overall increase in the daily ration offered to REX. ICOD was used as a proxy measure for WtP in sessions, as these animals live in enclosures that include natural fauna, such as fish and crustaceans. Thus, ICOD days are not necessarily representative of the total caloric intake of the animals as MMP animals are sometimes observed supplementing their diets with wild prey that pass through their enclosures, but rather are a proxy for animal interest in training sessions.

The increase in the size of the offered diet for ANK and JCK is likely a reflection of their age at the time of data collection, as young sea lions will consume more fish as they mature [70]. The increase in quantity of fish (in lbs.) fed to REX, as well as his decrease in Δ_D_ and reduction in ICOD and sick days (Table 2), suggested an overall improvement in health status for this animal. SLD’s decrease in offered diet quantity could be due to his older age and slowing metabolism but could also be reflective of a reduction in his practice of rumination (i.e., regurgitation and reingestion of food), thereby requiring fewer kilocalories to maintain a healthy weight. One means of stabilizing an animal that exhibits rumination syndrome is to increase the quantity of food received to minimize caloric losses due to stereotypic behavior [81,82]. Thus, SLD’s diet decrease, and subsequent increase in consuming his offered diet as well as sharp reduction in clinically ill days, suggest an improvement in his health and welfare and a possible decrease in the expression of his rumination behavior.

It is worth noting that all animals’ health remained high during the entire study period. SLD experienced periods of acute illness during Time Period 1 that amounted to 25 days (0.03% of the days in those two years), a value that declined to a single day in the two years of Time Period 2. Additionally, ANK was not clinically ill during the entire four-year period, and only experienced seven ICOD days during those years. Advances in animal management of marine mammals in professional facilities have resulted in extended life spans for California sea lions [83]. Targeted enrichment programs have reduced maladaptive and stereotypic behaviors (e.g., [48,49]) and computerized systems have been successful in lessening the frequency of such behaviors in some individual primates [58]. The introduction of EVE was successful in improving long-term health metrics in the focal individuals of this study.

Due to animal interest in EVE, the system is often used to elicit participation in sessions following instances of uninterest in training sessions or separating from a social group. While the level of interest in the system appears to vary by individual, disinterest in EVE sessions for animals that generally show a high level of focus and engagement while playing with EVE might serve as a future indicator for changes in animal health. However, potential confounding factors, such as observations of temporary animal disengagement on more difficult tasks, need to be considered.

EVE sessions have also been used as a means to reinforce valuable husbandry-related behaviors, such as voluntary venipuncture (i.e., blood draw). Preventative medicine programs with professionally managed animals have been important for continued progress in the veterinary field, with operant conditioning predicted to play a large role in the future advancements in the care of animals [30]. The successfulness of such a program largely depends on regular sample collections, and the voluntarily participation of trained animals results in safer and less stressful experiences [22,30]. Husbandry training has resulted in improved life expectancy for several marine mammal species in professional care, including California sea lions [83]. The application of highly reinforcing consequences following such behaviors supports the future success of the behavior, and trainers at the MMP have utilized EVE as a means to provide variable reinforcement opportunities following important training milestones. For example, one sea lion had not provided a voluntary blood draw during his annual physical for four years prior to being introduced to EVE. Since EVE has become a part of his regular enrichment plan, he has given voluntary blood samples twice a year, with the opportunity to interact with EVE used as reinforcement immediately following the completion of the behavior.

The length of EVE sessions is comparable to what MMP animals normally experience in other types of in-pen sessions (e.g., husbandry), and shorter than other sessions (e.g., open-water training). Thus, the average session length of the animals was not impacted, and the number of training sessions conducted in a day was not increased. Rather, EVE was used as another training session type within the same parameters of other in-pen training sessions and has been used as a shorter component of longer, open-water sessions (e.g., Figure 1). Previous pilot data utilizing an automated feeder (i.e., paddle operated and with EVE) found that the sea lions showed increased anticipatory behaviors prior to sessions that were trainer-guided (i.e., Trainer Only and Trainer with EVE; [84]). Subsequently, trainer-guided sessions are most frequently implemented when interacting with EVE. Additionally, once the sea lions mastered the Cursor Training Game [79], the type of game provided varies across sessions in order to prevent habituation and add additional cognitive challenge.

Anecdotally, trainers have mentioned that setting up and running EVE has been successful in encouraging animals to participate in sessions where they had been hesitant or disinterested in separating from the social group. Measuring animal interest in the system, both under stimulus control and outside of trainer-guided sessions and in the absence of primary reinforcement (i.e., an assessment of intrinsic motivation), would be informative to understand a baseline of the reinforcement value and WtP in EVE sessions. WtP has successfully been used as a predictor for changes in bottlenose dolphin health status [69] and should be evaluated as a potential predictor for sea lion health status.

## 5. Conclusions

This study provides evidence that the introduction of a computerized system had a positive impact on the welfare of US Navy sea lions when incorporated during training sessions and used in rotation with other standard forms of enrichment at the program. The integration of EVE into routine training sessions enhanced the participation and dietary consumption for focal animals. Additionally, a notable decrease in the incidence of illness was observed. These outcomes highlight the potential of cognitive enrichment to improve the health and overall welfare of marine mammals, supporting the notion that such interventions can serve as valuable components of animal management strategies in professional settings.

## Figures and Tables

**Table 1:** Focal subject classification and dates utilized.

Animal	Age at Start of Time Period 1	Classification	Time Period 1 Dates	Time Period 2 Dates	EVE Sessions in Time Period 2
ANK	9	EVE–Control	22 July 2018–22 July 2020	23 July 2020–23 July 2022	93
JCK	12	Non-EVE–Control	20 March 2018–20 March 2020	21 March 2020–21 March 2022 *	0
REX	15	EVE–Focal	8 September 2018–8 September 2020	9 September 2020–9 September 2022	183
SLD	16	EVE–Focal	2 August 2018–2 August 2020	3 August 2020–3 August 2022	113

* JCK began learning to interact with EVE on 22 March 2022. Thus, his four-year time period was selected to accommodate that date.

**Table 2:** Information for each individual’s number of days analyzed, days removed due to illness, number of ICOD days, as well as diet based on each two-year time period.

Subject	Time Period	Days Removed Due to Illness	Number of Days Analyzed	Days of ICOD	Average Diet (lbs)	Mean % of Diet Consumed
ANK	1	0	747	6	12.19	99.83
	2	0	743	1	13.04	99.93
JCK	1	0	728	6	11.58	99.89
	2	19	701	8	12.47	99.86
REX	1	5	717	61	15.19	97.36
	2	0	717	18	16.30	99.27
SLD	1	25	701	40	15.16	98.06
	2	1	721	15	13.23	99.37

## Data Availability

Data available upon reasonable request.
